# Seasonal Energetic Stress in a Tropical Forest Primate: Proximate Causes and Evolutionary Implications

**DOI:** 10.1371/journal.pone.0050108

**Published:** 2012-11-28

**Authors:** Steffen Foerster, Marina Cords, Steven L. Monfort

**Affiliations:** 1 Department of Ecology, Evolution and Environmental Biology, Columbia University, New York, New York, United States of America; 2 Institute of Primate Research, National Museums of Kenya, Nairobi, Kenya; 3 Smithsonian Conservation Biology Institute, Front Royal, Virginia, United States of America; German Primate Centre, Germany

## Abstract

Animals facing seasonal variation in food availability experience selective pressures that favor behavioral adjustments such as migration, changes in activity, or shifts in diet. Eclectic omnivores such as many primates can process low-quality fallback food when preferred food is unavailable. Such dietary flexibility, however, may be insufficient to eliminate constraints on reproduction even for species that live in relatively permissive environments, such as moist tropical forests. Focusing on a forest-dwelling primate with a flexible diet (*Cercopithecus mitis*) we investigated whether females experience seasonal energetic stress and how it may relate to reproductive seasonality. We used fecal glucocorticoids (fGCs) as an indicator of energetic stress, controlling for the potentially confounding effects of social interactions and reproductive state. We modeled within-female fGC variation with General Linear Mixed Models, evaluating changes in feeding behavior and food availability as main effects. Regardless of reproductive state, fGCs increased when females shifted their diet towards fallback foods (mature leaves and other non-preferred items) and when they spent more time feeding, while fGCs decreased with feeding time on preferred items (insects, fruits, young leaves) and with the availability of young leaves. Changes in fruit availability had no general effects on fGCs, likely because fruits were sought out regardless of availability. As predicted, females in the energetically demanding stages of late pregnancy and early lactation showed greater increases in fGCs between periods of low versus high availability of fruits and young leaves than females in other reproductive states. Potential social stressors had no measurable effects on fGCs. Preliminary evidence suggests that seasonal energetic stress may affect the timing of infant independence from mothers and contribute to unusually long inter-birth intervals compared to closely related species of similar body size. Our findings highlight how the study of stress responses can provide insights into the proximate control of reproductive strategies.

## Introduction

Despite minimal seasonal variation in day length and temperature, most tropical habitats experience seasonal fluctuation in rainfall that mediates changes in food availability for many animals. These environmental changes require behavioral and physiological adaptations to minimize negative effects on fitness [1], [2]. Among mammals in particular, natural selection should favor timing of reproduction relative to food availability to minimize energetic stress on females, whose reproductive success depends greatly on available energy reserves [3], [4]. Indeed, changes in food availability limit reproduction to specific times of year even in moist tropical forests [5], effects likely to be regulated by neuroendocrine pathways linking energy balance to production of gonadotropin-releasing hormone [6]. As seasonal reproduction may lengthen interbirth intervals and generally slow down reproductive rates, one may expect the evolution of phenotypic traits that reduce the impact of environmental seasonality on fitness.

Many primates are known especially for having evolved considerable dietary flexibility, most likely as a response to the challenge of environmental seasonality [7]. They are able to cope with periods of food shortage by exploiting alternative, non-preferred items, known as fallback foods [8], which are often lower than preferred foods in usable energy content and/or higher in plant secondary compounds. Although they have not been well studied in other mammals, the importance of fallback foods for primates has led researchers to view them as a driving force in the evolution of life histories, anatomy and behavior [9].

Many cercopithecine monkeys (e.g., macaques, baboons, guenons) are generalist feeders particularly well known for their resource switching capacity, which results from long gut retention times and hindgut fermentation that facilitates the digestion of fiber-rich foods [10]. In most populations of baboons, for example, behavioral and physiological adaptations have been successful enough to allow females to breed at any time of year even in extremely seasonal environments [11]. For other species, like most forest guenons (*Cercopithecus* spp.), the ability to rely on fallback foods to meet energetic demands has not eliminated seasonal constraints on reproduction, even when living in a tropical moist rainforest [12], [13]. Suggested proximate constraints limiting reproduction to certain times of year among primates include energetic stress in pregnant and lactating females [14]–[16], limited availability of weanling food [13], [17], temperature stress in newborn infants [18]–[20], or a combination of these and other factors [12].

It is well known that sufficient energy reserves in female vertebrates are required to trigger ovulation and successfully reproduce [3], [6], [21]. Actual energetic constraints, however, are unknown for most wild primate populations because energy balance is not easily quantified in wild animals. This also means that direct evidence for a selective pressure on the evolution of seasonal birth patterns is generally lacking [22]. Correlations between the timing of births and total plant food availability or rainfall, as used in past studies of birth seasonality [13], [23]–[26], cannot easily be interpreted as indicators of energetic constraints because they do not quantify food quality or energy expenditure in relation to intake. An improved understanding of the adaptive value of reproductive strategies may be achieved by investigating the physiological mechanisms that directly influence reproduction [27], [28].

The hypothalamic-pituitary-adrenal axis can exert control over individual reproductive decisions through its regulation of the stress response system. Glucocorticoids (GCs) are the main mediators of physiological stress responses in vertebrates [29], and one of the main functions of GCs is to optimize energy availability [30]. This makes GCs useful indicators of physiological responses to changes in food availability [31], [32], food intake [33], [34], and feeding effort [35], [36] that can provide valuable insights into the selective forces acting on the evolution of behavioral and life history strategies [37]–[39]. While variation in GC levels has been related to the timing of reproduction, parental investment, and breeding success in birds [40]–[43], the relationship between stress hormones and reproduction appears complex among mammals, varying by species, type of stressor, intensity, duration, or the interaction with reproductive hormones [44]. So far, only few studies have explicitly investigated the relationship between environmental stressors and reproductive strategies in wild primates [28], [45]–[47], and studies on female primates are particularly rare [11], [48]. Here, our primary goal was to investigate how temporal variation in physiological stress responses may help us understand the proximate mechanisms that mediate female reproductive strategies in a tropical forest monkey, using as an example a well-studied population of blue monkeys (*Cercopithecus mitis stuhlmanni*) in western Kenya [18].

Assessing the causes and consequences of environmental seasonality in a wild social vertebrate such as blue monkeys is complicated by the fact that GC production can be influenced by many factors [29]. For example, seasonally recurring energetic stress can be associated with other energetic challenges such as mating activity or reproductive state [45], [49], [50], while agonistic interactions within social groups may lead to psychological stress [51], [52] that can confound changes in energetic stress. Therefore, identifying factors that may constrain the timing of female reproduction must include the simultaneous assessment of multiple potential stressors known to influence GC production in social vertebrates, of which we investigated the following:

### Reproductive State

Because of placental secretion of corticotrophin-releasing hormone in non-human primates [53], we expected females to excrete elevated fGC levels in the second half of pregnancy relative to other reproductive states, all else being equal. Independent of this proximate cause of within-individual fGC variation, environmental and social factors can present additional stressors that modulate fGC changes within females over time. If such additional stressors cause relatively large changes in GCs over time when compared to GC changes stemming from females changing reproductive states, the within-individual patterns of GC excretion over time may not primarily be related to reproductive state and may exist in most individuals regardless of individual differences in absolute GC levels at any one time.

### Potential Energetic Stressors

Changes in feeding efficiency as a result of variable food availability and levels of feeding effort may present considerable energetic challenges that influence female reproductive strategies. Relative changes in feeding on specific food items of high versus low energetic quality can be informative and allow inferences about changing energetic challenges [54], even if total time spent feeding may be a relatively poor indicator of food intake [55]. Therefore, as females increase their feeding time on food items that are comparatively rich in usable energy, like insects, ripe fruits, and young leaves, levels of energetic stress are expected to decrease. Conversely, the greater reliance on mature leaves low in usable energy as well as indicators of feeding effort (i.e., energy spent searching for food) may be associated with energetic stress [56], [57]. In the presence of energetic stressors, we predicted that females in the energetically most demanding states of reproduction, late pregnancy and early lactation, would be particularly sensitive to changes in food availability, responding to energetic stressors with greater changes in fGCs than females in other reproductive states.

### Potential Psychological Stressors

Negative social interactions such as agonism can trigger psychological stress, while affiliative social interactions can reduce the magnitude and duration of stress responses to potential psychological stressors [58], [59]. Therefore, the influence of these social interactions on fGCs needs to be assessed and potentially controlled for before making conclusions about the existence of energetic challenges. Agonism did not explain temporal fGC variation in *C. m. albogularis* [54] and did not contribute to individual differences in fGCs among the study females [60]; hence we did not expect that rates of agonism would influence temporal fGC variation. However, we previously found evidence that grooming directed at others was associated with lower individual mean fGC levels after controlling for agonism and feeding on fruits [60], indicating that differential coping opportunities may modulate stress responses. Whether grooming received or given is more important for buffering stress responses is unclear, as both have been associated with lowering GCs [61], [62].

### Climate

Although seasonal variation in temperature can be a significant metabolic stressor that affects GC excretion in wild primates [11], [48], [63], temperature variation is minimal across months in the Kakamega Forest given its location near the equator (mean monthly temperature range 1997–2006∶19.9–22.5°C; [64]). It is possible, however, that seasonal variation in rainfall could interact with the cool night-time temperatures to drive seasonal variation in temperature stress, e.g., when monkeys experience cool nights after heavy afternoon rains. Rainfall could also influence stress responses indirectly through its effects on food availability. However, as the relationship between rainfall and primate food availability is highly variable in tropical forests [65], it is difficult to predict exactly how fluctuations in rainfall might relate to the quantity and quality of food available to blue monkey females.

## Methods

### Ethics Statement

This study was purely observational, and all study animals were well habituated to human observation prior to data collection. The non-invasive collection of fecal samples for hormone analyses did not interfere with the natural behavior of the study animals. All data collection protocols were approved by Institutional Animal Care and Use Committee of Columbia University (IACUC 2885, 4307, 7232), and the Kenyan Ministry of Environmental Science and Technology (Permit #: 13/001/35C 252). All research was undertaken in accordance with the ABS/ASAB guidelines for the ethical treatment of animals in research and teaching.

### Study Site and Subjects

The study population inhabits the Isecheno study site of the Kakamega Forest, western Kenya (0° 14′ 11″ N, 34° 52′ E, elevation 1618 m), a semi-deciduous rainforest with annual rainfall averaging approximately 2000 mm. Rain falls seasonally, with the dry season typically spanning December to March, and a shorter period of low rainfall in June/July [66]. Several groups of blue monkeys have been observed since 1979 [18].

Females in this population give birth primarily between January and March [18], a period that overlaps with a seasonal peak in fruit availability (January/February; Cords, unpublished data). A study on infant development in this population showed that infants markedly decrease daytime suckling and contact time with the mother after the third month of life, and move about largely independently by the age of six months [67]. Although it is difficult to determine the exact timing of a peak in maternal energetic demand [68], it is likely that this period occurs when infants have maximal growth rates and high activity levels, yet still rely largely on milk [69]. We estimate this period of peak lactation to lie between the 3^rd^ and 5^th^ month of an infant’s life, similar to what has been suggested for rhesus and Japanese macaques [69], [70].

Subjects for this study were 21 adult females from two study groups, GN (10 females of 35–39 total members that included 10–12 adult females and 4–7 infants) and TWS (11 females of 43–52 total member that included 12–14 adult females, and 1–11 infants) that occupied adjacent home ranges (about 20 and 31 hectares, respectively). Agonistic interactions are rare in this population. Group-wide rates of agonism varied from 0.1 to 0.4 interactions per hour in GN (mean: 0.2, N = 6 monthly means across female means), and from 0.2 to 0.7 interactions per hour in TWS (mean: 0.4). Nevertheless, a linear hierarchy among adult females is well established [71], [72]. Agonism occurs primarily over food (73% of all agonism with identified context during the study period [60], 82% of all agonism [71]). Fruits were the most contested of all food items during the study period (47% of all food-related agonism) and they were contested disproportionately to their representation in the diet (30% of time spent feeding across 21 females).

### Data Collection

#### Behavioral sampling

SF conducted 922 30-minute focal samples on all females in six non-consecutive months (with 1 or 2-month gaps) between September 2005 and October 2006. All females were observed in the same months for about equal lengths of time. Total observation time per female was 23.0±0.9 hours (range: 21.5–24.5 hours), and averaged 3.8±0.2 hrs per month. During each follow, SF recorded all activity states continuously with an approximate accuracy of ±3 seconds, using a handheld computer (Workabout MX, Psion Teklogix, Inc., Missisauga, Ontario) and Observer Software (Noldus Information Technology, Inc., Leesburg, VA). Feeding included manipulating and ingesting food items, but not simply chewing food from filled cheek pouches. Agonistic interactions were recorded on an all occurrence basis, with partner identity and context (food, space, grooming partner, infant, and other) whenever possible (74% of 431 interactions). To assess energy expenditure for travel, the distance covered during each individual movement was estimated visually to the nearest meter and then summed for each focal sample.

#### Food availability

A field assistant (W. Adukha) assessed phenology of feeding trees at bi-weekly intervals between September 2005 and October 2006 by surveying a random selection of trees (5–20 per species depending on their abundance in the habitat; diameter at breast height, DBH ≥10 cm) from 25 known important food species ([73]; personal observation) distributed throughout both home ranges. We surveyed up to 4 specimens for an additional 19 species that were rarely eaten and/or rare in the study area. Adukha estimated abundance of young leaves and leaf buds (LV), flowers and flower buds (FL), and unripe and ripe fruits/seeds (FR) on a scale of 0–4 (4 = 100% of crown coverage). To measure tree abundance we established a total of 77 randomly placed 20 m×20 m vegetation plots (GN: 30, TWS: 47), covering about 6% of the home range of each group. We measured the DBH of all trees with DBH ≥10 cm. We used these measurements to estimate the total basal area (BA) of each species in the home range based on a stratified sampling method considering three habitat types: main forest (old growth), woodland (secondary growth), and forest station (scattered trees). For each food item in each home range, we then obtained a food availability score (FA) by multiplying its average biweekly phenology score with its total estimated BA [74]. We averaged FA scores across two surveys per month to obtain a mean monthly FA score for each item. We summed FA scores across items to index total monthly food availability, and used the proportion of this total food availability accounted for by a specific food class to evaluate whether it was consumed disproportionately.

For the food types most often consumed (fruits: 30% of all feeding time, across females and months; young leaves: 31% of feeding time), we calculated availability scores in two ways, first by summarizing across all species eaten (total availability), and second by summarizing across species that were eaten for at least 1% of the time spent feeding, on average, across all females and study months (main item availability). For flowers and ‘other’ food items, only three species met our criteria of ‘main’ food items (flowers: *Celtis africana* in GN and *Morus mesozygia* in TWS; ‘other’: *Cupressus lusitanica* cones in both groups); for these two food items we therefore considered only total availability estimates in our analyses. However, as the pollen-filled male cones of *Cupressus lusitanica* accounted for an average of 81.7± SE 0.2% of feeding time on ‘other’ items (N = 6 monthly means across females), any effects of ‘other’ food types in our analyses is likely driven by this particular food item. Conversely, the two main flower items did not account for a disproportionate amount of feeding time on flowers, allowing only general inferences about the importance of flowers from our models. We ignored mature leaves in calculating availability scores because they changed little in availability.

#### Fecal sample collection and processing

SF and field assistants collected 3,322 fecal samples over 14 months between September 2005 and October 2006, of which 1,683 came from 10 females in GN and 1,639 from 11 females in TWS (12± SD 4 samples per female per month). Within a median of 87 minutes after defecation (range: 2–393 minutes; 98% within 180 minutes) we transferred fecal samples to a camping oven (Coleman Company, Inc., Wichita, KS, model no. 5010D700T) and dried them at 90±5°C. We removed samples from the field oven once per day in the evening, resulting in a mean drying time of 5.6±2.0 hrs. For our analyses, we excluded samples that dried for less than two or more than nine hours (2.1% of samples). Samples were stored in the dark at ambient temperatures (∼25°C) for a median duration of 35 days (range: 2–91) before they were transferred to a freezer (−20°C) for long-term storage. Immediate drying followed by freezing is generally regarded as one of the best preservation methods for fecal steroids [75], [76] and once dried and frozen, sample concentrations are stable for up to three years or more (Barrette, Young, and Monfort, in prep.).

We quantified fGCs in the Endocrine Research Laboratory of the Smithsonian Conservation Biology Institute, Front Royal, VA. Detailed extraction procedures are described elsewhere [54]. Average extraction efficiency per batch was 84.7% (range: 72.4–97.6%, N = 24). Inter-assay coefficients of variation (CV) were 10.3% and 9.9% for high and low assay kit controls, respectively (N = 20), while inter-assay CVs for a low, medium, and high blue monkey sample run with each assay were 11.3%, 8.4% and 6.5%, respectively. All samples were run in duplicates; intra-assay CV for a mixed fecal pool averaged 8.5% (N = 14). We determined GC concentrations using a ^125^I-double-antibody radioimmunoassay (MP Biomedicals, Orangeburg, NY, catalog no. 07-120103) as described previously [77], which we validated for *C. mitis* in a prior study by showing GC responses to an ACTH challenge [54].

### Statistical Analyses

#### Confounding factors

We evaluated the effects of potential confounds on sample fGC measurements, including time of day, sample storage time, and fiber content, using multivariate linear mixed models (Text S1). The best model indicated that fGCs in individual samples decreased with time of day, an effect that we confirmed in post-hoc analyses using paired samples collected from the same females on the same day at different times. There were no differences in mean sample times among females in any given month, or among months across females, suggesting that monthly mean fGCs were not biased by the timing of sample collection. To validate this assumption we re-ran our main models using monthly means derived from residuals of a model controlling for time of day, and found no changes in the direction or relative magnitude of effects. A second predictor in the best model indicated that fGCs increased with fiber content, which we assessed with a semi-quantitative measure of fiber strands in dried crushed fecal samples. Although a high fiber diet generally accelerates gut passage times, increases fecal volume and ultimately decreases fecal GC concentrations in wet samples, drying of fecal samples controls for this effect [78]. Hence, the weak positive effect of fiber on fGCs is likely to indicate dietary changes rather than altered gut passage times. We tested the contribution of fiber content as an indirect indicator of mature leaf consumption when modeling temporal fGC variation.

#### Model selection

For all behavioral variables, we calculated average proportions and frequencies across all focal observations for a given month to obtain a monthly mean for each female. Months included in the models are those for which we had both detailed behavioral and environmental data: October 2005, January, March, June, August, and October 2006. We excluded a given female from the analysis in any month when she was observed <5 times (N = 3) or when <3 fecal samples were available (N = 3) to achieve a minimum level of reliability of estimates. To assess the influence of reproductive state, we determined the pregnancy period for each female post-hoc from the birth of an infant, assuming a gestation period of 176 days [79]. For each day of fecal collection, we assigned one of the following reproductive states: second half of pregnancy (a period of hypercortisolism), first six months of lactation (elevated energetic demand of lactation and infant carrying), and all other reproductive states. We then averaged across all sampling days to arrive at the predominant reproductive state for a given female and month.

We used General Linear Mixed Models to analyze temporal patterns in our longitudinal data, because they allow for subject-specific deviations from mean temporal response patterns, correlated errors of measurement within individuals, and the optimization of covariance structures [80]. We assessed the determinants of temporal fGC variation using monthly mean concentrations per female as the dependent variable. All models included female identity as a random factor modeled with a variance component (independent) covariance structure, month as a repeated measure, and predictor variables as fixed factors. To remove between-group effects from our longitudinal analyses we centered both fGCs and predictor variables on their respective group means (grand mean across all months). Based on model fit criteria (see below), we determined that a first-order autoregressive heterogeneous covariance structure was the most appropriate covariance structure for modeling our repeated measures.

We entered reproductive state as a categorical fixed effect, and all other predictors as continuous covariates. As behavioral predictor variables for within-female variation in fGCs over time (i.e., across months) we included: time spent feeding on fruits, flowers, young leaves, mature leaves, insects, and other food items; fiber content; time spent grooming; rate of agonism received plus given; time spent moving and distance moved per focal observation. As environmental factors, we entered rainfall as well as estimates of the availability of main food items in the following categories: fruits, flowers, young leaves. We started with a full model including all hypothesized predictors, including first-order interactions of covariates with reproductive state, and removed parameters, one by one, that were least likely to explain fGC variation in the data until we arrived at a best model given the data. We assessed model fit with a small sample size correction of the Akaike Information Criterion (AIC_C_) that approaches AIC values with larger sample sizes, and compared the relative fit of alternative models using Akaike weights and evidence rations [81]. We considered a model for inference if its evidence ratio (the odds against being the true best model given the data, relative to the best model) was less than ten. We used SPSS 20.0 for Windows (SPSS Inc., Chicago, IL) for all analyses.

## Results

### Temporal Variation of Food Availability

**Figure 1:**
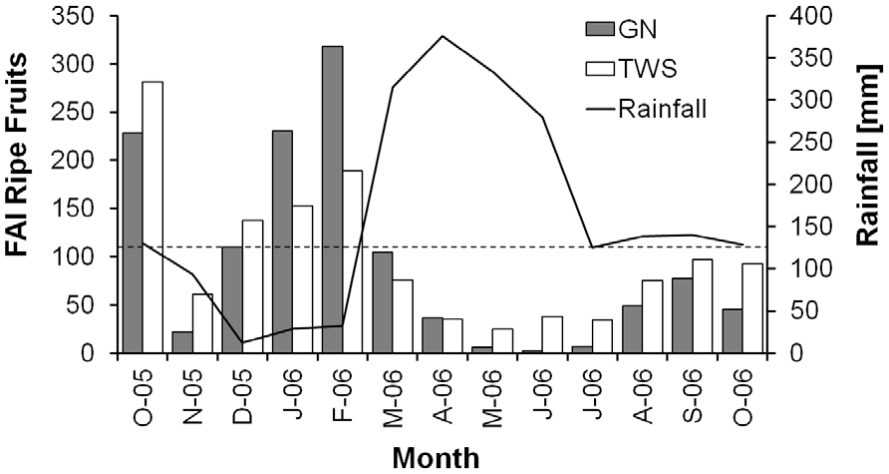
Fruit availability and rainfall during the study period. Temporal change in food availability index (FAI) for main ripe fruits, eaten by adult female blue monkeys for at least 1% of all feeding time across months in study groups TWS and GN, and monthly rainfall recorded during the study period. Dashed horizontal line shows mean fruit availability across all months and groups.

**Figure 2:**
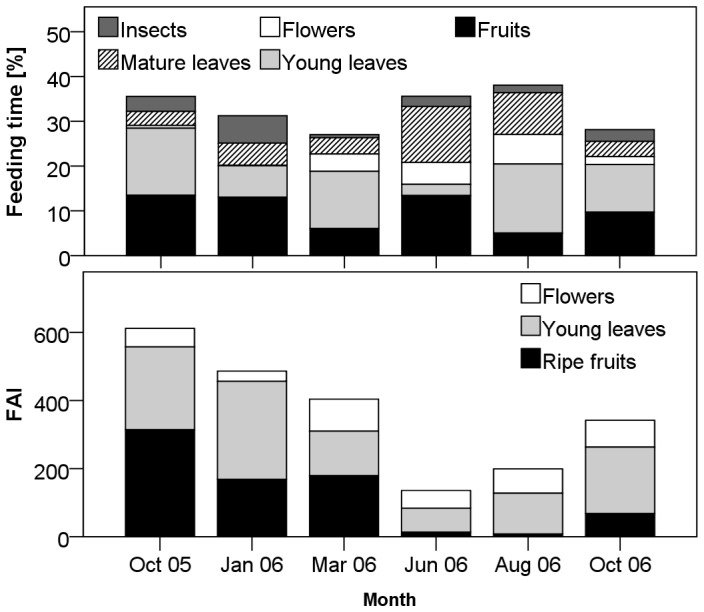
Time devoted to feeding and availability of main food items. Temporal variation in mean percentage of observation time spent feeding on different food types (top), and mean monthly food availability index (FAI) for the three most commonly eaten food types (bottom).

For main fruit items (those eaten for >1% of feeding time across females and months), fruit availability decreased temporarily in November/December 2005, and was generally low from April 2006 until the end of the study period (Figure 1). However, one of the most important food species, the non-indigenous *Bischofia javanica*, showed a peak in fruiting in June 2006, at which time feeding on fruits was almost entirely focused on this one species. Largely because of the availability of *B. javanica*, feeding time on fruits remained relatively high in June despite the lowest habitat-wide availability of fruits (Figure 2). Given the selective feeding on fruits, monthly group averages of time spent feeding on fruits were not correlated with our estimates of ripe fruit availability (Spearman rank correlation: r_S_ = 0.22, p = 0.48, N = 12 monthly group averages). Young leaves, in contrast, may have been eaten in proportion to availability (r_S_ = 0.57, p = 0.055), suggesting less selective feeding on this food type compared to fruits. Overall, main fruit items made up about 5 times more of the diet than expected based on their combined availability, while main young leaf items accounted for about 2 times more of the diet than expected. Thus, ripe fruits are the most highly sought out plant food items, suggesting that they play an important role for meeting nutritional demands of female blue monkeys. Ripe fruit and young leaf availability were both negatively correlated with the time spent feeding on mature leaves (r_S_ = −0.71, p = 0.009 and r_S_ = −0.63, p = 0.028, respectively; N = 12 monthly group averages) and other food items (r_S_ = −0.6, p = 0.039 and r_S_ = −0.59, p = 0.042), indicating that mature leaves and other food items can be considered non-preferred fallback foods [8].

### Determinants of Within-Individual fGC Variation

**Figure 3:**
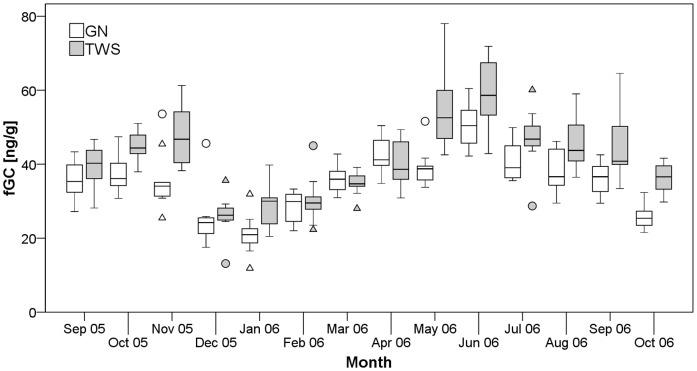
Temporal variation in fGC excretion across females in two study groups. Horizontal lines are medians, boxes show inter-quartile range (IQR), whiskers indicate 95% confidence intervals, circles mark outliers more than 1.5x IQR, and triangles mark extreme outliers more than 3x IQR.

There were pronounced fluctuations in fGC levels that were similar in both study groups (Figure 3). This temporal pattern of fGC excretion was not primarily related to reproductive status; females that were neither pregnant nor lactating during the study period (N = 9) showed a temporal pattern of fGC variation that was indistinguishable from females that were pregnant or lactating (N = 11), as indicated by the absence of an interaction effect between reproductive state and month across 14 months from which we had fGC data (F_2, 73.9_ = 0.97, p = 0.38). Although comparisons of pregnant vs. non-pregnant, non-lactating females within each month did show the expected between-subject effect of pregnancy on absolute levels of fGC excretion (Figure S1), females showed similar relative variation in their fGCs over time that were not simply a result of changes in reproductive state alone (Figure S2).

Our best model given the data predicted within-female variation in fGCs with reproductive state, feeding behavior, and variation in food availability (Table 1). All else being equal, females during their second half of pregnancy and first six months of lactation had higher fGC concentrations than females in other reproductive states. Additional increases in fGCs were associated with time spent feeding on non-preferred fallback food items such as mature leaves and “other” items (mainly represented by *C. lusitanica* cones), and with total time spent feeding. In contrast, fGCs were relatively low in months when females spent more time feeding on insects. Neither time spent moving nor distance moved were associated with fGC changes (see Table S1, for model fits including these factors), suggesting that increases in fGCs were not caused by females spending more energy on searching for food. Further, temporal variation in the rates of agonistic interactions or time spent grooming were not related to temporal changes in fGC excretion (Table S1). In addition to feeding behavior, mean monthly fGC levels were elevated when availability of flowers and ‘other’ food items was higher than average, and fGC levels increased further with rainfall. Greater availability of young leaves (main food items) was associated with lower fGCs, while changes in fruit availability had no independent effects on within-female variance in fGC levels in this best model (see Table S2, for model fits including this factor). Fiber content of feces, although documented to influence individual sample GC concentrations (Text S1), did not independently explain monthly variation in fGCs within females.

**Table 1:** Parameter estimates of the best General Linear Mixed Model explaining variation in monthly female-mean fGCs in two groups of blue monkeys as a function of changes in reproductive state, feeding behavior, food availability estimates, and rainfall.

Parameter^1, 2^	Estimate	SE	df	95% Confidence Interval	
				Lower	Upper
Intercept	−6.50	1.06	57.72	−8.62	−4.38
Second half of pregnancy^3^	11.36	2.43	50.50	6.47	16.24
First six months of lactation^3^	4.24	1.28	48.36	1.66	6.82
Feeding on insects	−0.55	0.16	43.48	−0.88	−0.22
Feeding on mature leaves	0.22	0.10	68.12	0.02	0.43
Feeding on other fallback items	0.28	0.12	57.80	0.05	0.52
Time spent feeding	0.17	0.05	75.46	0.07	0.27
Availability of young leaves	−0.04	0.01	79.29	−0.06	−0.02
Availability of flowers	0.12	0.01	99.66	0.10	0.15
Availability of food items other than fruits, flowers, or young leaves^4^	0.66	0.09	93.61	0.48	0.84
Rainfall	0.02	0.01	38.94	0.01	0.03

1 All measures expressed relative to group mean across months (N = 6).

2 For alternative models, see Table S1, Table S2, and Table S3.

3 Compared to females in other reproductive stages.

4 Primarily male cones of *Cupressus lusitanica.*

An equally well fitting but less parsimonious model contained all variables included in the best model described above and two additional variables related to feeding behavior (Table S3). In this model, time spent feeding on fruits and time spent feeding on young leaves were both negatively related to fGCs. Dropping either one of these two variables resulted in slightly worse model fits (Table S3). Models that considered only variables related to either food availability or feeding behavior, but not both in combination, received substantially less support (Table S1, Table S2).

**Figure 4:**
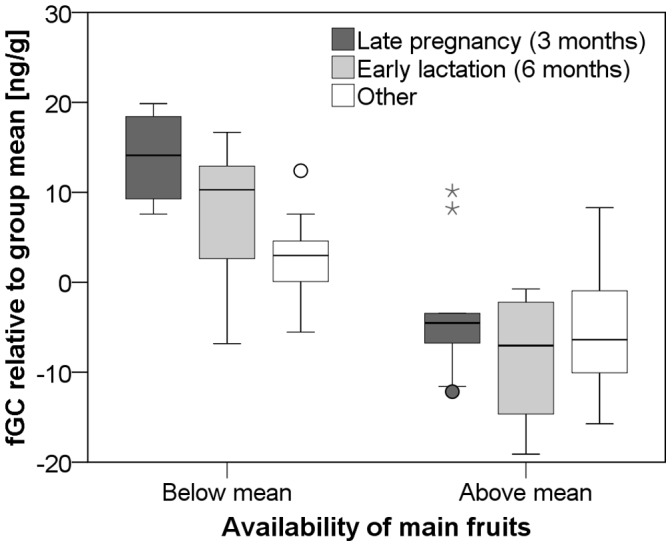
Effect of female reproductive state and ripe fruit availability on fGC excretion. Boxplot of fGC excretion in periods of below- and above-average availability of ripe fruits (main items) for females of different reproductive states. Horizontal lines are medians, boxes show inter-quartile range (IQR), whiskers indicate 95% confidence intervals, circles mark outliers more than 1.5x IQR, and triangles mark extreme outliers more than 3x IQR.

**Figure 5:**
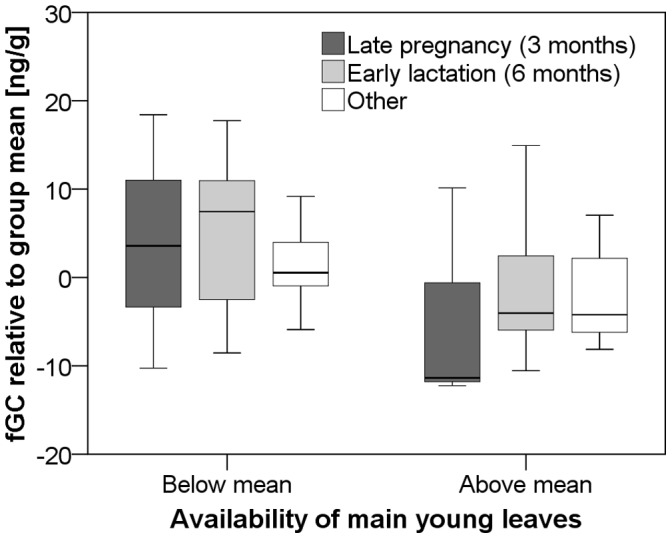
Effect of female reproductive state and young leaf availability on fGC excretion. Boxplot of fGC excretion in periods of below- and above-average availability of young leaves (main items) for females of different reproductive states. Horizontal lines are medians, boxes show inter-quartile range (IQR), whiskers indicate 95% confidence intervals, circles mark outliers more than 1.5x IQR, and triangles mark extreme outliers more than 3x IQR.

To test our prediction that females would be particularly sensitive to changes in food availability while they were in the energetically challenging stages of late pregnancy and early lactation, we compared fGC levels within females of a given reproductive state between periods of below- and above average food availability across all 14 study months (Figure 4). We found that the difference in fGC levels between periods of below- and above-average availability of ripe fruits was significant for females of all reproductive states, but that effect size was greatest for females during late pregnancy and early lactation (Figure 4; late pregnancy: 17.3± SE 2.9 ng/g, z = −2.2, p = 0.028, N = 6; early lactation: 14.2± SE 2.3 ng/g, z = −2.4, p = 0.018, N = 7; other: 7.4± SE 1.8 ng/g, z = −3.0, p = 0.002, N = 20; Wilcoxon signed-rank tests). Similarly, we found that the difference in fGC between periods of below- and above-average availability of young leaves was 3–4 times greater for females who were in the stages of late pregnancy and early lactation compared to other females (Figure 5: late pregnancy: 8.4± SE 3.7 ng/g, z = −1.6, p = 0.11, N = 3; early lactation: 6.9± SE 2.3 ng/g, z = −2.3, p = 0.026, N = 11; other: 2.9± SE 1.3 ng/g, z = −2.2, p = 0.027, N = 21; Wilcoxon signed-rank tests).

**Figure 6:**
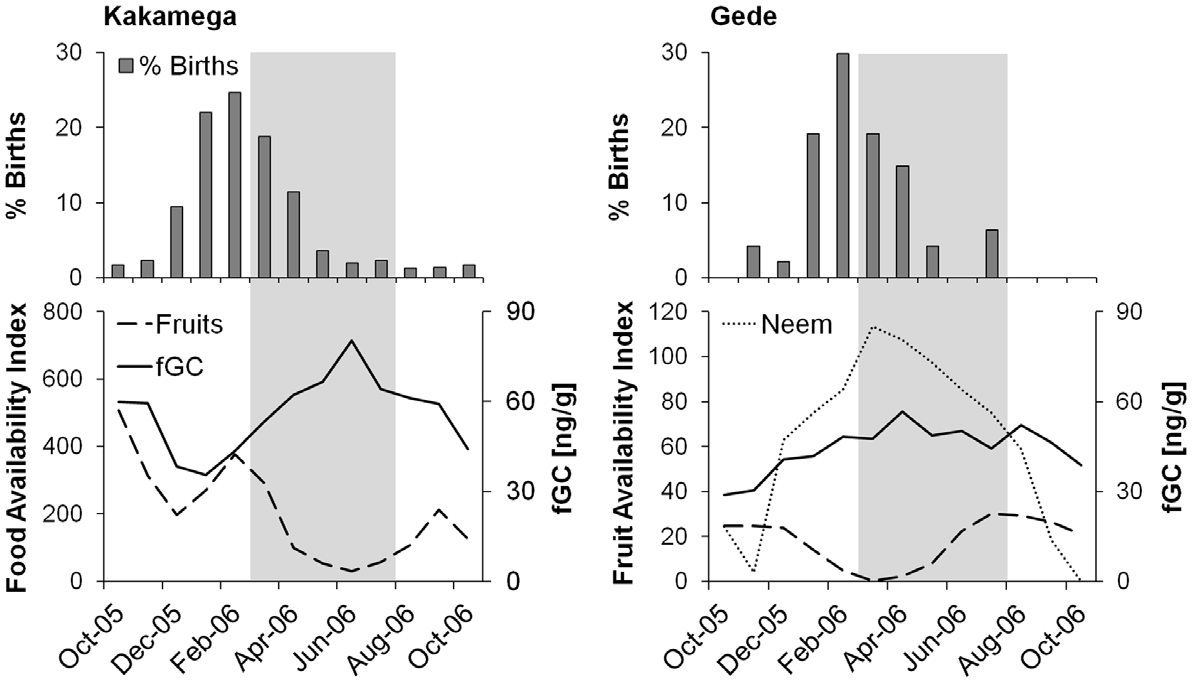
Comparison of birth seasonality, fruit availability, and fGC excretion in two populations of *Cercopithecus mitis.* Distribution of births (N = 347 for *C. m. stuhlmanni* [18]; 47 for *C. m. albogularis* (Foerster and Wahungu, unpublished data), availability of fruits, and fGC excretion in a study population of *C. m. stuhlmanni* (Kakamega) and *C. m. albogularis* (Gede). Data on fruit availability and fGC excretion come from a one-year study period in both populations (2005–2006). Peak lactation period (shaded box) is set at 3–5 months after the birth peak [67], [69]. At Gede, neem (*Azadirachta indica*) is a very common non-preferred fruit eaten by monkeys when preferred fruits are rare, but clearly associated with increased feeding effort that influences fGC excretion [54]; in Kakamega, no non-preferred but widely eaten fruit items are available during the low-fruit season.

## Discussion

Our study assesses the importance of potential energetic challenges in the lives of female monkeys using physiological measures of stress responses, with the goal of better understanding the adaptive value of reproductive seasonality in a rainforest environment. We found that after controlling for reproductive state, an increase in the availability of non-preferred food items as well as increases in the total feeding time and in feeding on non-preferred food items (mature leaves, other fall back items) was associated with an increase in fGC levels, despite considerable dietary flexibility in the study species and year-round availability of fallback food items. In contrast, increased feeding on foods relatively rich in easily digestible energy, such as insects, fruits, and young leaves, was associated with lower fGCs, as was the availability of young leaves that were generally more preferred than mature leaves, flowers, and other non-fruit items. Females who were in the stages of late pregnancy and early lactation were more sensitive to variation in the availability of preferred food sources, i.e. ripe fruits and young leaves, and showed greater increases in fGCs between periods of high versus low availability of these items compared to females in other reproductive states. We found no evidence that social interaction in a daily life context mediated fGC changes over time for a given female.

The results presented here indicate that temporal variation in GCs can be a useful tool to monitor variable energetic stress in female blue monkeys, a conclusion supported by a number of studies on wild social vertebrates. Decreases in GC levels coincided with periods of high fruit consumption in chimpanzees [31] and female ring-tailed lemurs [33], and were associated with increased availability of preferred fruits and food supplements in wild Sykes’ monkeys [54]. Studies of other mammals, such as squirrel monkeys, elephant seals, and African elephants, have also shown that periods of increased feeding effort and/or food shortage (including fasting) can lead to GC elevations [34], [82], [83]. In contrast, changes in reproductive state, often seen as a main cause of temporal GC variation in female primates [84]–[86], were not a primary determinant of temporal fGC variation in female blue monkeys. Regardless of reproductive state, all females showed roughly the same temporal pattern of fGC excretion, which suggests that this variation was primarily determined by external stressors. In food limited populations, this finding should not be surprising, as reproductive physiology is only one among many causes of both individual and temporal variation in stress responses. Detailed studies of birds, for example, have documented how environmental factors such as food availability continue to influence GC production during reproduction, in addition to the changes brought about by fetal development and parental investment [87]. Our results support the importance of considering the role of external stressors, along with reproductive state, in influencing longitudinal changes in GCs among wild female primates.

The small but significant positive effect of rainfall on fGC variation is difficult to interpret. In some environments, rainfall may be a good indirect indicator of plant food availability and may show a negative temporal correlation with GC levels [11], [47], as may temperature changes [63] and behavioral time budgets [48]. As the effects of rainfall on fGCs in female blue monkeys persisted after controlling for food availability estimates, and because more rainfall was related to higher fGCs, perhaps females experienced some degree of cold stress during cool nights in the rainy season. It is also possible that variation in rainfall was related to unknown changes in food availability that influenced foraging efficiencies and energy balances.

Contrary to a population of conspecifics, indicators of feeding effort (i.e., time spent moving and distance moved) did not have measurable effects on fGC variation in female blue monkeys. Among Sykes’ monkeys at Gede Ruins, Kenya, high fGC excretion coincided with periods of significantly elevated feeding effort in the absence of more preferred energy-rich fruit resources [54]. Greater dietary diversity in the Kakamega Forest may have buffered females from more extreme fluctuations in feeding efficiency; at Gede, only 7 food items were eaten for >1% of feeding time (across months and females), while at Kakamega this list included 26 and 24 identified items for GN and TWS respectively. It is also possible that our estimates of time spent moving and distances moved were not sufficiently reliable to reflect changes in energy expenditure, perhaps because we did not distinguish between horizontal and vertical movements.

Temporal changes in rates of agonism, which in this population relates primarily to contest competition over fruits [60], [71], did not have a measureable effect on fGC levels. We recently documented the same lack of an effect in closely related *C. mitis albogularis* [54]. Moreover, we found no relationship between individual mean rates of agonism and individual mean fGCs in our study population [60]. By contrast, some primate field studies have reported a significant positive association between individual rates of agonism and GCs (*Papio anubis*: [88], *Lemur catta*: [89], *Propithecus verreauxi*: [90]). The reason for the inconsistent effects of agonism across studies and organisms may be that agonism influences fGC variation only when it is related to a high level of unpredictability about the nature and quality of social relationships and social status [86], or when it occurs in the absence of sufficient coping opportunities [52], [91]. Aggressive conflict over food in a daily life context may not result in prolonged physiological stress responses under natural, stable social conditions, leaving energetic stress as the major correlate of temporal as well as individual GC variation [31], [54].

Although there is some uncertainty associated with using feeding times to estimate relative changes in food intake [55] and our assessment of food availability is incomplete (no data on non-tree food items), changes in feeding time on particular food types (e.g., fruits, leaves, flowers, insects) are likely to reflect changes in energy assimilation if variation in feeding efficiency is, on average, greater between than within food types. In addition, the absence of any evidence for an influence of potential psychological stressors on fGCs, as well as similar conclusions obtained from two prior studies on *C. mitis* [54], [60], lead us to believe that the temporal variation in fGCs that we observed indeed reflected energetic stress in periods of increased reliance on fallback food, and improved energetic status when females were able to obtain more of their preferred foods.

### Implications for Life History Evolution

Across guenon populations in different parts of Africa, peak fruit abundance appears to coincide with the birth peak and earliest weeks of lactation, while the energetically most demanding period of peak lactation often occurs when fruits are less abundant [13]. The same pattern appears to hold for our study population, where births peak during a period of relatively high fruit availability, as judged by the plant phenology data collected during our study period (similar seasonal patterns emerge across additional years of data from multiple groups; Cords, unpublished data). However, while it was suspected that variation in fruit availability causes seasonal energetic challenges, these challenges have never been measured directly, and it therefore remained unclear whether the seasonal environmental changes were indeed the proximate causes of seasonal reproduction in these rainforest primates. After all, constraints other than food availability may influence the timing of reproduction, including rainfall and its possible effect on temperature regulation in infants that may influence survival chances among blue monkeys and others [18], [92].

The data on physiological responses to changes in food availability presented here indicate directly that female guenons experience seasonal energetic challenges, a finding that can inform our understanding of their reproductive strategies, and may relate to the evolution of exceptionally slow life history in blue monkeys [18]. Slow life histories have been reported in other forest-dwelling primates, while related species inhabiting more open, seasonal, and less predictable habitats reproduce earlier and at higher rates [93]. Among African monkeys, species differences in life history “speed” remain even under captive conditions [94]. Such differences in life history speed may be a response to differences in age-specific mortality [95]; higher adult mortality in open habitats should favor earlier maturity and higher birth rates, while lower adult mortality in tropical forests should favor later maturity and lower birth rates. Comparative studies have supported this framework for wild primates (e.g., [96]), and adult mortality in this blue monkey population is indeed very low compared to close relatives with faster life histories [18]. Mortality, however, interacts in complex ways with ecological factors to cause variation in life histories traits [97]; life history speed in mammals can vary within and between species as a function of food availability [98], [99], an effect that can be independent of mortality rates (birds: [27], ungulates: [100], [101], primates: [102]). For example, chacma baboons (*P. ursinus*) exhibited significantly longer interbirth intervals in a highly seasonal than in a less seasonal environment, and the proximate cause of this difference was thought to be variability in food resources that constrain both infant independence and female reproduction [103].

Assessing the physiological responses to ecological variability and how these responses influence reproductive decisions [38], [39] can give us insights into the ecological constraints that influence species-typical life history strategies. The seasonal energetic stress we documented for female blue monkeys suggests that energetic constraints may not only influence the timing of reproduction, but also contribute to long inter-birth interval in this population (30 months when the first infant survives at least 12 months; [18]). In our study population, females that gave birth in a given year are likely to have infants that are 4–7 months old as the next mating season arrives (mid-June through October; [104]). At this age, infants increase physical activity yet still depend on mothers for food and partially for transport [67], [105]. Therefore, lactating females are likely to experience the highest energetic demand – peak lactation [69], [106] – shortly before or during the mating season that follows the birth of their infant. As conception is influenced by body condition [6], only females without young infants may be in sufficiently good body condition to conceive during this time, which also overlaps the period of lowest fruit availability.

Preliminary comparative evidence supports this interpretation. In *C. mitis albogularis* (Gede Ruins, Kenya; [54]), 83% of 47 births occurred between January and April (Foerster and Wahungu, unpublished data). Availability of fruits in this semi-deciduous lowland forest was relatively high during much of the peak lactation period (Figure 6) as well as the main mating season (July to October). Further, low-intensity food supplementation by humans provided additional preferred food (e.g., bananas) from December to March and July to September [54]. The more consistent fruit availability at Gede, as well as higher availability of fruits during peak lactation and conception coincided with less seasonal variation in GC excretion and a considerably shorter interbirth interval (mean: 18.1± SD 4.5 months, N = 24 intervals in which first infant survived at least one year) compared to females at Kakamega (30.7±9.3 months, N = 193; [18]).

Energetic stress among lactating females may influence another noteworthy aspect of the life history of blue monkeys, the relatively fast development of infant independence from mothers [67]. While low risk of predation or conspecific threat to infants may *allow* early independence [67], the energetic stress of lactating females may actually *drive* it. Females excreted peak fGC levels in May-June, when most infants were 4–5 months old (average age on June 1∶4.5 months, range: 2.2–7.7 months, N = 15), i.e. near the end of what we estimated as the period peak lactation. As the availability of preferred fruits was low and females instead relied more heavily on fallback foods, mothers had an energetic incentive to encourage infant independence, thus safeguarding their own health. Perhaps it is not a coincidence that allomaternal care of infants also peaks at around the same infant age, facilitated by mothers leaving their infants in the care of older juveniles [105]. Although multiple functions of allomaternal care in this and other species may exist, independent locomotion of infants is likely to increase foraging efficiency of the mother [17] and may be a crucial component of adaptation to seasonally changing environmental conditions.

In conclusion, we documented significant environmental stressors in the lives of tropical forest guenons and provided preliminary evidence that these stressors may have implications for female reproduction. As such, our study reveals a potential proximate mechanism that may have mediated life history evolution in blue monkeys and other forest guenons. We believe that further studies integrating behavioral, ecological, and hormonal measures can lead to a better understanding of the evolution of life history variation among extant vertebrates. Given the extent of physiological and behavioral variation across species as well as intra-specific variability based on environmental and social factors, however, it is not likely that there will be a single unifying explanation for how different populations and species respond to stressful stimuli. Careful investigation of multiple potential stressors in each population is therefore necessary to interpret the adaptive significance of GC variation.

## Supporting Information

Figure S1 **Effect of reproductive state on individual differences in fGC excretion.** The figure shows fecal GC excretion in pregnant (grey bars) and non-lactating, non-pregnant females (white bars) during the four months preceding the peak of the birth season. The numbers above the x-axis indicate sample sizes. Females were included only if more than five fecal samples were available for a given month and reproductive stage. Horizontal lines are medians, boxes encompass interquartile range (IQR), and whiskers connect lowest and highest values excluding outliers. Outliers up to 1.5x IQR are indicated by circles, extreme outliers more than 3x IQR are indicated by triangles. * p<0.05; ** p<0.01.
(PDF)

Figure S2 **Temporal variation in mean fGC excretion in 21 female blue monkeys.** Each line shows the temporal change in fGC concentrations, relative to the group mean, for one female. Relative changes over time during the study period are similar among females regardless of their reproductive states.
(PDF)

Table S1 **List of General Linear Mixed Models testing hypotheses about the causes of monthly fGCs variation.** Models include reproductive state, feeding behavior, social interactions, and fiber content, for six non-consecutive months. Shown are multivariate models with non-zero fixed effects as well as univariate models for all predictors (controlling for reproductive state). Evidence ratios give the odds against a given model being the best model, given the data and the best model in the set.
(PDF)

Table S2 **List of General Linear Mixed Models testing hypotheses about the causes of monthly fGCs variation.** Models include reproductive state, estimates of food availability (FA), rainfall, and fiber content, for six non-consecutive months. Shown are multivariate models with non-zero fixed effects as well as univariate models for all predictors. Evidence ratios give the odds against a given model being the best model, given the data and the best model in the set.
(PDF)

Table S3 **List of General Linear Mixed Models testing hypotheses about the causes of monthly fGCs variation.** Models include the best behavioral and environmental predictors identified in Tables S1 and S2. Evidence ratios give the odds against a given model being the best model, given the data and the best model in the set.
(PDF)

Text S1 **Assessing the influence of confounding factors on sample fGCs.**
(PDF)
