# Supplementary material for: Seasonal Energetic Stress in a Tropical Forest Primate: Proximate Causes and Evolutionary Implications
Source: PLoS One. 2012 Nov 28;7(11):e50108. doi: 10.1371/journal.pone.0050108 (PMC3509155; doi:10.1371/journal.pone.0050108)

Supporting Information: "Seasonal energetic stress in a tropical forest primate: proximate causes and evolutionary implications"

Steffen Foerster, Marina Cords, Steven L. Monfort

Figure S1: Effect of reproductive state on individual differences in fGC excretion

The figure shows fecal GC excretion in pregnant (grey bars) and non-lactating, non-pregnant females (white bars) during the four months preceding the peak of the birth season. The numbers above the x-axis indicate sample sizes. Females were included only if more than five fecal samples were available for a given month and reproductive stage. Horizontal lines are medians, boxes encompass interquartile range (IQR), and whiskers connect lowest and highest values excluding outliers. Outliers up to 1.5x IQR are indicated by circles, extreme outliers more than 3x IQR are indicated by triangles. * $p<0.05$; ** $p<0.01$

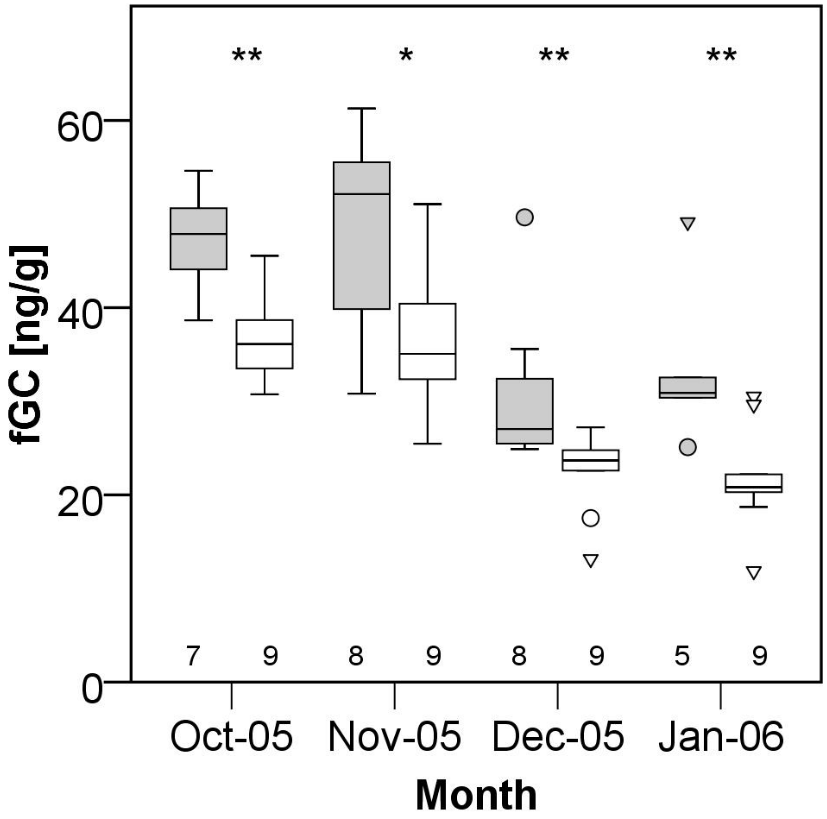

Supplement: Figure S1 — Effect of reproductive state on individual differences in fGC excretion. The figure shows fecal GC excretion in pregnant (grey bars) and non-lactating, non-pregnant females (white bars) during the four months preceding the peak of the birth season. The numbers above the x-axis indicate sample sizes. Females were included only if more than five fecal samples were available for a given month and reproductive stage. Horizontal lines are medians, boxes encompass interquartile range (IQR), and whiskers connect lowest and highest values excluding outliers. Outliers up to 1.5x IQR are indicated by circles, extreme outliers more than 3x IQR are indicated by triangles. * p<0.05; ** p<0.01. (PDF) [file pone.0050108.s001.pdf]
