# Supplementary material for: Seasonal Energetic Stress in a Tropical Forest Primate: Proximate Causes and Evolutionary Implications
Source: PLoS One. 2012 Nov 28;7(11):e50108. doi: 10.1371/journal.pone.0050108 (PMC3509155; doi:10.1371/journal.pone.0050108)

Supporting Information: **"Seasonal energetic stress in a tropical forest primate: proximate causes and evolutionary implications"**

Steffen Foerster, Marina Cords, Steven L. Monfort

Figure S2: Temporal variation in mean fGC excretion in 21 female blue monkeys

Each line shows the temporal change in fGC concentrations, relative to the group mean, for one female. Relative changes over time during the study period are similar among females regardless of their reproductive states.

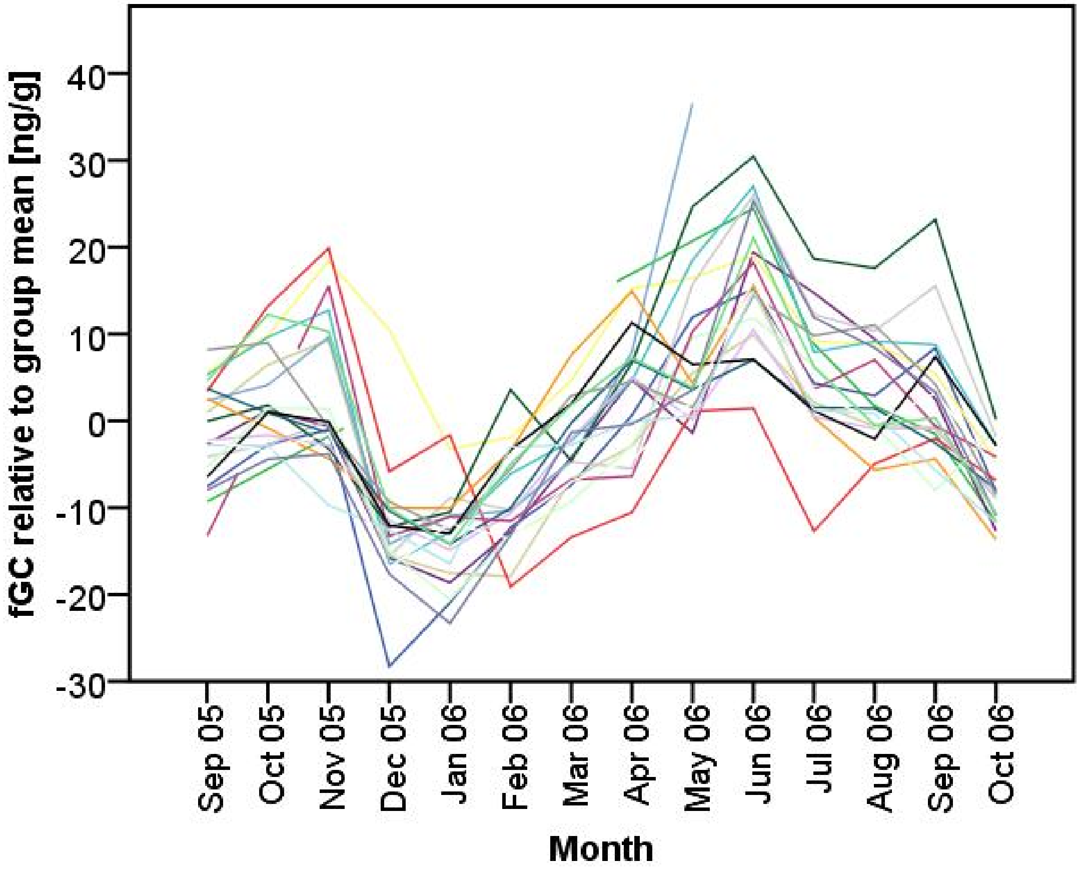

Supplement: Figure S2 — Temporal variation in mean fGC excretion in 21 female blue monkeys. Each line shows the temporal change in fGC concentrations, relative to the group mean, for one female. Relative changes over time during the study period are similar among females regardless of their reproductive states. (PDF) [file pone.0050108.s002.pdf]
