# Supplementary material for: Seasonal Energetic Stress in a Tropical Forest Primate: Proximate Causes and Evolutionary Implications
Source: PLoS One. 2012 Nov 28;7(11):e50108. doi: 10.1371/journal.pone.0050108 (PMC3509155; doi:10.1371/journal.pone.0050108)
Supplement: Table S1 — List of General Linear Mixed Models testing hypotheses about the causes of monthly fGCs variation. Models include reproductive state, feeding behavior, social interactions, and fiber content, for six non-consecutive months. Shown are multivariate models with non-zero fixed effects as well as univariate models for all predictors (controlling for reproductive state). Evidence ratios give the odds against a given model being the best model, given the data and the best model in the set. (PDF) [file pone.0050108.s003.pdf]

Supporting Information: “Seasonal energetic stress in a tropical forest primate: proximate causes and evolutionary implications”

Steffen Foerster, Marina Cords, Steven L. Monfort

Table S1: List of General Linear Mixed Models testing hypotheses about the causes of monthly fGCs variation.

Models include reproductive state, feeding behavior, social interactions, and fiber content, for six non-consecutive months. Shown are multivariate models with non-zero fixed effects as well as univariate models for all predictors (controlling for reproductive state). Evidence ratios give the odds against a given model being the best model, given the data and the best model in the set.

Model AIC_C	Intercept	Reproductive state	Time feeding on fruits	Time feeding on mature leaves	Time feeding on young leaves	Time feeding on flowers	Time feeding on insects	Time feeding on other	Time feeding	Rate of agonism	Time grooming	Time moving	Distance moved per follow	Fiber content	Evidence Ratio
805.1	x	x		x			x	x	x					x	1
812.4	x	x					x	x	x					x	38
816.4	x	x					x	x						x	293
817.6	x	x		x			x	x							528
818.5	x	x		x			x	x	x						816
850.1	x	x						x							5999848165
850.1	x	x		x											6121053138
853.6	x	x					x								34183195479
853.9	x	x								x					40924715155
854.8	x	x										x			62285843140
856.6	x	x								x	x				156293267620
857.1	x	x													201690463465
858.3	x	x				x									369346106731
858.5	x	x			x										394151030919
859.5	x	x											x		662972932164
859.8	x	x	x												785825032788
859.9	x	x									x				801699751477
870.5	x														163854507399628
