# Supplementary material for: Seasonal Energetic Stress in a Tropical Forest Primate: Proximate Causes and Evolutionary Implications
Source: PLoS One. 2012 Nov 28;7(11):e50108. doi: 10.1371/journal.pone.0050108 (PMC3509155; doi:10.1371/journal.pone.0050108)
Supplement: Table S2 — List of General Linear Mixed Models testing hypotheses about the causes of monthly fGCs variation. Models include reproductive state, estimates of food availability (FA), rainfall, and fiber content, for six non-consecutive months. Shown are multivariate models with non-zero fixed effects as well as univariate models for all predictors. Evidence ratios give the odds against a given model being the best model, given the data and the best model in the set. (PDF) [file pone.0050108.s004.pdf]

Supporting Information: “Seasonal energetic stress in a tropical forest primate: proximate causes and evolutionary implications”

Steffen Foerster, Marina Cords, Steven L. Monfort

Table S2: List of General Linear Mixed Models testing hypotheses about the causes of monthly fGCs variation.

Models include reproductive state, estimates of food availability (FA), rainfall, and fiber content, for six non-consecutive months. Shown are multivariate models with non-zero fixed effects as well as univariate models for all predictors. Evidence ratios give the odds against a given model being the best model, given the data and the best model in the set.

Model AIC_C	Intercept	Reproductive state	FA Fruits (main items)	FA Fruits	FA Young leaves (main items)	FA Young leaves	FA Flowers	FA Other items	Rainfall	Fiber content	Evidence Ratio
806.3	x	x			x		x	x	x		1
808.0	x	x			x		x	x		x	2
813.0	x	x									29
816.0	x	x		x	x		x	x			128
819.8	x	x		x		x	x		x		851
859.0	x								x		277753537039
859.6	x				x						374928058308
868.0	x					x					25002576612851
869.3	x			x							47893456332463
870.5	x										87267567199066
871.9	x		x								175735299721479
873.9	x						x				477698071851700
875.1	x							x			870422637631299
