# Supplementary material for: Seasonal Energetic Stress in a Tropical Forest Primate: Proximate Causes and Evolutionary Implications
Source: PLoS One. 2012 Nov 28;7(11):e50108. doi: 10.1371/journal.pone.0050108 (PMC3509155; doi:10.1371/journal.pone.0050108)
Supplement: Table S3 — List of General Linear Mixed Models testing hypotheses about the causes of monthly fGCs variation. Models include the best behavioral and environmental predictors identified in Tables S1 and S2. Evidence ratios give the odds against a given model being the best model, given the data and the best model in the set. (PDF) [file pone.0050108.s005.pdf]

Supporting Information: "Seasonal energetic stress in a tropical forest primate: proximate causes and evolutionary implications"

Steffen Foerster, Marina Cords, Steven L. Monfort

Table S3: List of General Linear Mixed Models testing hypotheses about the causes of monthly fGCs variation.

Models include the best behavioral and environmental predictors identified in Tables S1 and S2. Evidence ratios give the odds against a given model being the best model, given the data and the best model in the set.

Model AIC$_C$	Intercept	Reproductive state	FA Fruits (main items)	FA Young leaves (main items)	FA Flowers	FA Other items	Rainfall	Time feeding on fruits	Time feeding on mature leaves	Time feeding on young leaves	Time feeding on flowers	Time feeding on insects	Time feeding	Evidence Ratio
792.8	x	x		x	x	x	x					x	x	1
792.8	x	x		x	x	x	x	x		x		x	x	1
794.7	x	x		x	x	x	x	x				x	x	3
794.7	x	x		x	x	x	x			x		x	x	3
798.9	x	x	x	x	x	x	x					x	x	21
801.3	x	x		x	x	x	x		x			x		70
803.4	x	x		x	x	x	x		x	x		x		199
805.1	x	x		x	x	x	x	x	x	x		x		466
805.1	x	x		x	x	x	x	x	x	x	x	x		466
806.3	x	x		x	x	x	x							850
