# Supplementary material for: Seasonal Energetic Stress in a Tropical Forest Primate: Proximate Causes and Evolutionary Implications
Source: PLoS One. 2012 Nov 28;7(11):e50108. doi: 10.1371/journal.pone.0050108 (PMC3509155; doi:10.1371/journal.pone.0050108)
Supplement: Text S1 — Assessing the influence of confounding factors on sample fGCs. (PDF) [file pone.0050108.s006.pdf]

Supporting Information: "Seasonal energetic stress in a tropical forest primate: proximate causes and evolutionary implications"

Steffen Foerster, Marina Cords, Steven L. Monfort

Text S1: Assessing the influence of confounding factors on sample fGCs

We used general linear mixed models (GLMM) to analyze the effects of potential confounds on fGC concentrations, using all available samples collected between September 2005 and October 2006 (N=14 months). We square root transformed fGC concentrations to obtain normal distributions. We entered female identity as a random effect, day as repeated measure modeled with an autoregressive covariance structure, and potential confounds as covariates. In post-hoc analyses we assessed whether any confounding factors identified by the GLMM had systematic effects on monthly averages, which were the units of analysis in subsequent models of temporal variation.

Table A below identifies the models we ran to test the influence of potential confounds on fGC concentrations in individual fecal samples. Models are sorted by AIC_C in ascending order, and the intercept-only model is shaded. Evidence ratios are the ratio of Akaike weights of the best model and a given alternative model, i.e. the odds against a given model being the best model, given the data (and given the best model in the set). Table B gives parameter estimates of the three best models.

The best model (top row) identified two confounding variables: time of day and fiber content. Fecal concentrations of GCs decreased during the course of the day, while higher fiber content was associated with elevated fGC concentrations.

Pair-wise comparisons between morning (before 10:30 am) and afternoon (after 2:30 pm) samples from the same female collected on the same day confirmed this finding; there was a significant decrease in mean fGC from morning (38.0 ± 1.9 ng/g) to afternoon (31.8 ± 1.5 ng/g; $p<0.001$, N=62, paired t-test). However, a month-wise comparison of collection times across females did not indicate any significant between-subject effects in any month (ANOVA with time as dependent variable and female identity as fixed factor, Bonferroni adjustment for 14 tests, $p>0.0036$ for all months). Thus, although fGC concentrations decreased with time of day, the comparison of monthly averages was not systematically biased by the influence of this factor. Fiber content was still positively correlated with fGCs when using monthly group-wide means of fiber content and monthly mean fGC levels ($r_S=0.67$, $p<0.01$, N=14 monthly averages across 21 females). We therefore continued to assess the role of fiber content in subsequent models of monthly averages.

There were two additional models that were close in model fit (evidence ratio <10) and caused some model selection uncertainty. The second best model (ER=3.39) contained time of day as the only predictor variable, with an identical parameter estimate, while the third model indicated that the number of days in storage, before samples were frozen, was positively related to fGC levels after controlling for time of day and fiber content. Mean time in storage at ambient temperatures was 37.4 ± SE 0.4 days (N=3,075 samples), and each additional day in storage

increased fGC concentrations by 0.01 ng/g, all else being equal. There were no significant differences among females in sample storage durations (F=0.5, p=0.958) and standardized effect size was very small (r=0.05). Further, monthly changes in storage durations were not correlated with fGC concentrations (r_S=0.11, p=0.703, N=14 monthly means across female means). We concluded that storage of dried samples at ambient temperature had no systematic effects on fGC concentrations that could influence our temporal comparisons, and we did not further account for this factor in our temporal modeling.

Table A

#	Model AIC_C	Time of day	Fiber	Days to freezing	Seeds	Evidence ratio
1	9008.52	x	x			1.00
2	9010.96	x				3.39
3	9011.06	x	x	x		3.56
4	9047.63					91788722.55
5	9049.95			x		292799900.43
6	9050.82		x			452365029.83
7	9051.92				x	784063053.14

Table B

#	Parameter	Estimate[1]	SE	95% Confidence Interval	
				Lower Bound	Upper Bound
1	Intercept	9.31	0.17	8.98	9.64
	Time of day	-0.07	0.01	-0.09	-0.05
	Fiber	0.09	0.03	0.03	0.15
2	Intercept	9.34	0.17	9.01	9.68
	Time of day	-0.07	0.01	-0.09	-0.05
3	Intercept	9.09	0.18	8.73	9.46
	Fiber	0.09	0.03	0.03	0.15
	Time of day	-0.07	0.01	-0.09	-0.05
	Days to freezing	0.01	0.00	0.00	0.01
